# A Case of Distinctive Corneal Opacity Following Ciliary Sulcus Ahmed Tube Insertion in a Patient With Glaucoma Associated With Atopic Dermatitis

**DOI:** 10.7759/cureus.101272

**Published:** 2026-01-11

**Authors:** Chisako Ida, Hinako Ohtani, Keigo Takagi, Yuto Yoshida, Masaki Tanito

**Affiliations:** 1 Department of Ophthalmology, Shimane University Faculty of Medicine, Izumo, JPN

**Keywords:** ahmed glaucoma valve, atopic dermatitis, atopic glaucoma, ciliary sulcus tube insertion, corneal opacity, eye rubbing, tube shunt surgery

## Abstract

Corneal and conjunctival inflammation associated with atopic dermatitis is classified as atopic keratoconjunctivitis, and eye rubbing related to ocular itching is known to cause various ocular complications. We report a case of a Japanese man in his 40s with open-angle glaucoma associated with atopic dermatitis who developed a characteristic corneal opacity after Ahmed Glaucoma Valve (AGV) implantation with ciliary sulcus tube insertion. Despite the absence of persistent anatomical tube-corneal touch on serial examinations, a stellate corneal opacity developed at a site precisely corresponding to the tube tip during a period of exacerbated atopic dermatitis accompanied by frequent eye rubbing. Anterior-segment optical coherence tomography (OCT) consistently demonstrated a measurable distance between the tube and the corneal endothelium. Intermittent tube-corneal contact induced by corneal deformation during eye rubbing was hypothesized to be a plausible contributing factor. This case provides a clinical lesson that corneal damage related to the tube may occur even after sulcus tube insertion in patients with atopic glaucoma, highlighting the importance of careful postoperative corneal monitoring and appropriate management of atopic dermatitis.

## Introduction

Corneal and conjunctival inflammatory findings observed in patients with atopic dermatitis are classified as atopic keratoconjunctivitis [1]. Eye rubbing, which patients often perform to relieve eyelid itching or burning sensations, has been reported to be associated with various ocular disorders, including keratoconus, acute corneal hydrops, cataract, lens subluxation, intraocular lens dislocation, and rhegmatogenous retinal detachment [2-6]. Open-angle glaucoma associated with atopic dermatitis (atopic glaucoma) tends to be severe and difficult to manage, owing to its frequent association with atopic cataracts and retinal detachment, as well as chronic ocular surface and eyelid inflammation [7,8]. Tube-corneal touch is a known complication of tube shunt surgery performed for glaucoma [9]; however, when the tube is inserted into the ciliary sulcus, the distance between the tube and the cornea is generally longer than with anterior chamber insertion, and therefore, tube-corneal touch is generally uncommon [10].

In the present case, we encountered a patient with glaucoma associated with atopic dermatitis who developed a characteristically shaped corneal opacity after tube shunt surgery with sulcus tube insertion. Although a persistent anatomical tube-corneal touch was not observed on serial examinations, intermittent tube-corneal contact during episodes of eye rubbing may have contributed to the development of the corneal opacity.

## Case presentation

A Japanese man in his 40s had a medical history of hypertension and atopic dermatitis, for which he had long been using topical corticosteroids over the entire body. Fifteen years earlier, he visited a local ophthalmology clinic because of blurred vision in his left eye (OS). Markedly elevated intraocular pressure (IOP) exceeding 60 mmHg OS was detected, and antiglaucoma treatment was initiated. Subsequently, he underwent two trabeculotomies and one trabeculectomy OS. No corneal endothelial diseases, including Fuchs’ endothelial corneal dystrophy, were noted during follow-up.

**Figure 1:**
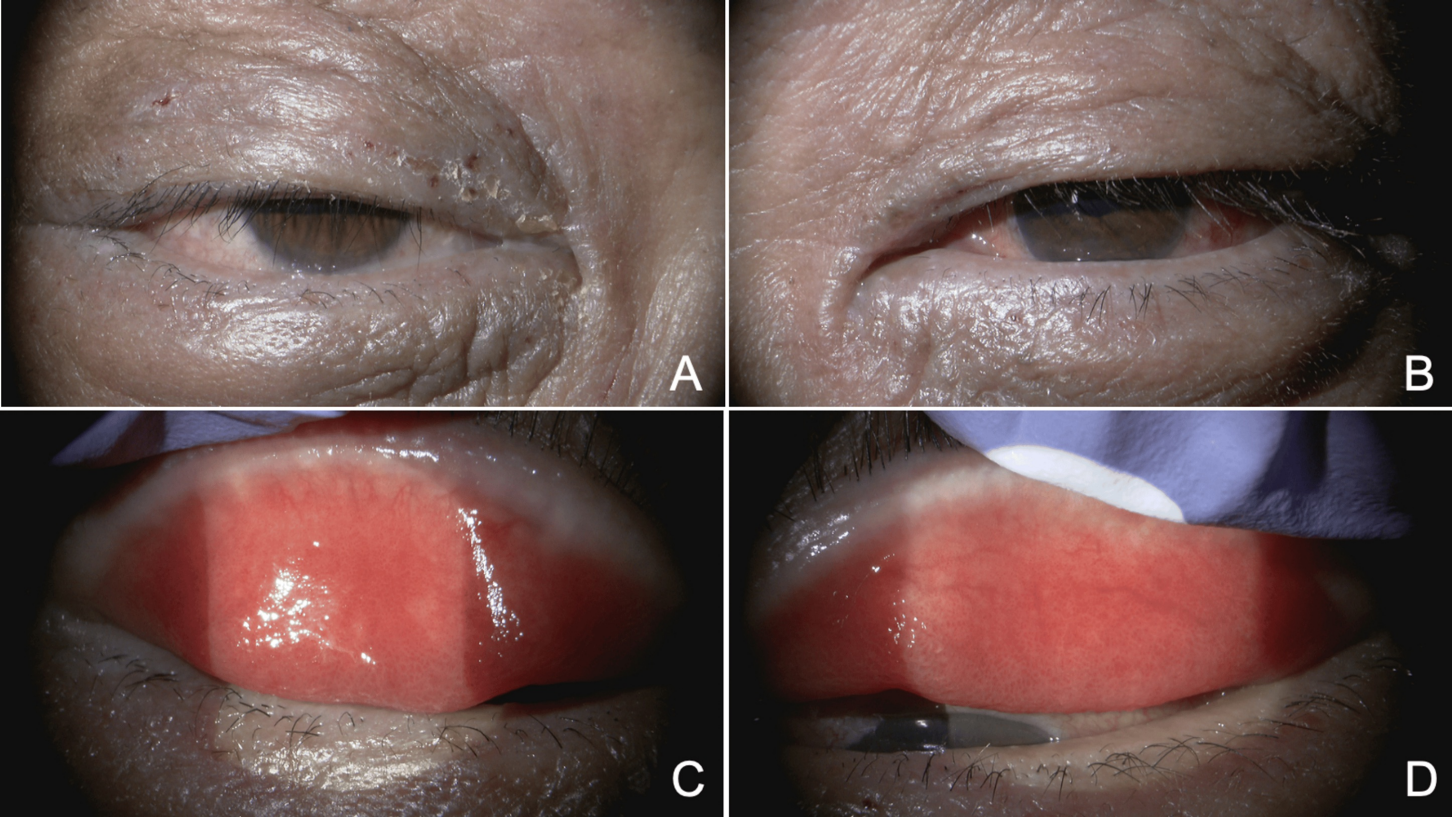
Anterior segment findings of the right eye (A, C) and left eye (B, D) (A, B) Eyelid dermatitis associated with atopic dermatitis is observed in both eyes. (C, D) Edematous changes and hyperemia of the palpebral conjunctiva are present in both eyes

One year after trabeculectomy, ophthalmic examination revealed a best-corrected visual acuity (BCVA) of 0.7 in the right eye (OD) and 0.15 OS. Goldmann applanation tonometry showed an IOP of 9 mmHg OD and 27 mmHg OS, with the OS receiving three antiglaucoma medications. The anterior chamber was deep OU without asymmetry. Gonioscopy [11] demonstrated Shaffer grade 4 open angles OU, with Scheie grade 1 pigmentation OU; scattered peripheral anterior synechiae related to previous glaucoma surgery were observed OS. A scarred filtering bleb was present superonasally OS. The crystalline lens was clear OD and showed mild cortical opacity OS. The vertical × horizontal cup-to-disc ratio was 0.7 × 0.7 OD and 1.0 × 0.9 OS, with no abnormal fundus findings other than glaucomatous changes. Marked scaling and crusting of the eyelid skin OU were observed (Figures 1A-1B). Both the bulbar and palpebral conjunctivae were edematous and hyperemic OU (Figures 1C-1D). Superficial punctate keratitis was present OU. No cornea guttata was observed in either eye. The patient was diagnosed with open-angle glaucoma OS associated with atopic dermatitis [7] and long-term use of topical corticosteroids applied extensively to the skin, as well as atopic blepharitis and conjunctivitis OU.

To improve IOP control OS, Ahmed Glaucoma Valve (AGV) implantation (FP-7, New World Medical, Irvine, CA) with simultaneous cataract surgery was planned. Preoperative serum IgE level was 7,156 IU/mL. For two months before surgery, latanoprost OS, timolol/dorzolamide OS, and topical epinastine and tacrolimus OU were prescribed. The AGV plate was placed in the superotemporal quadrant, and the tube was inserted into the ciliary sulcus using the micro-incision scleral tunnel (MIST) technique [12]. Cataract surgery was performed through a 2.2-mm nasal corneal incision, and an intraocular lens (+17.5 D, XY-1SP, HOYA Corporation, Tokyo, Japan) was implanted in the capsular bag. At the end of surgery, triamcinolone acetonide (15 mg) was injected around the plate. Postoperatively, 1.5% levofloxacin and 0.1% betamethasone phosphate eye drops were administered four times daily for three weeks. No shallow anterior chamber, overfiltration, or hypotony was observed during the postoperative period.

**Figure 2:**
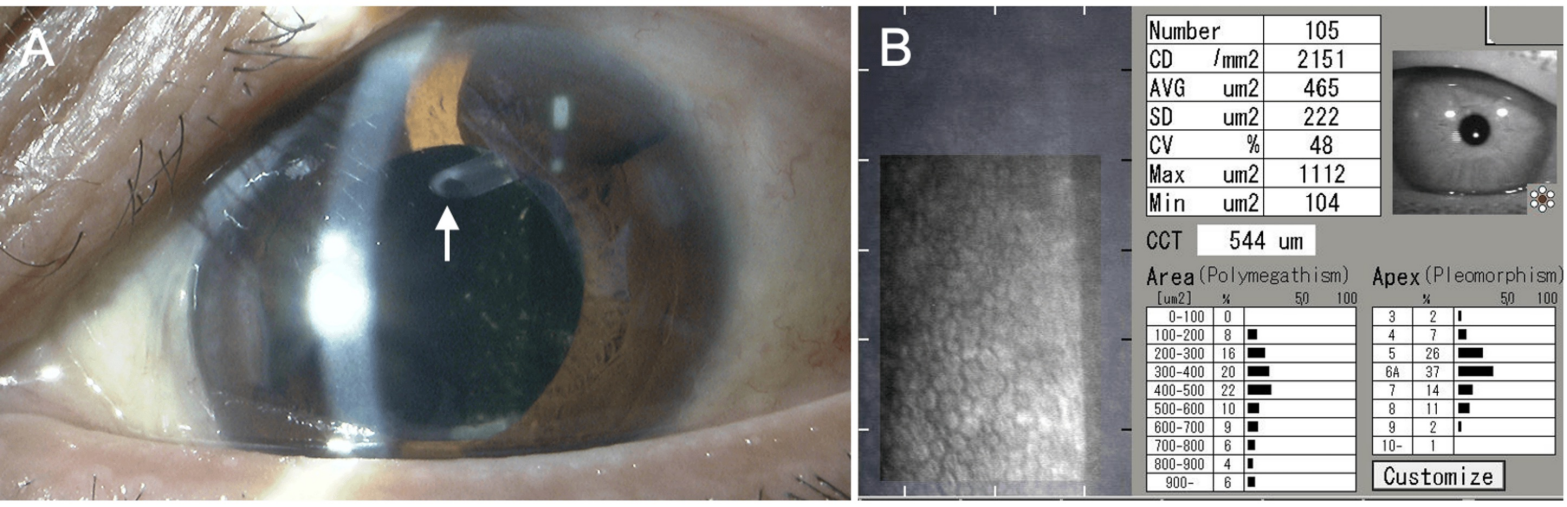
Slit-lamp (A) and specular microscopy (B) findings two months after Ahmed Glaucoma Valve implantation with ciliary sulcus tube insertion in the left eye CD: cell density; AVG: average; SD: standard deviation; CV: coefficient of variation; CCT: central corneal thickness (A) Adequate distance is maintained between the tube tip inserted into the ciliary sulcus (arrow) and the corneal endothelium. (B) Corneal endothelial cell density is 2,151 cells/mm², and central corneal thickness is 544 μm

At two months postoperatively, BCVA was 0.15 OS, and IOP was 8 mmHg OS. The anterior chamber remained deep, the tube position was appropriate, and sufficient distance was maintained between the tube and the corneal endothelium (Figure 2A, arrow). The mean deviation on the Humphrey 30-2 visual field test (Carl Zeiss Meditec, Dublin, CA) was -3.52 dB OD and -20.94 dB OS. Corneal endothelial cell density (ECD) was 2,151 cells/mm², and central corneal thickness (CCT) was 544 μm OS, measured by specular microscopy (EM-3000, Tomey, Nagoya, Japan). Only epinastine eye drops OU were continued.

**Figure 3:**
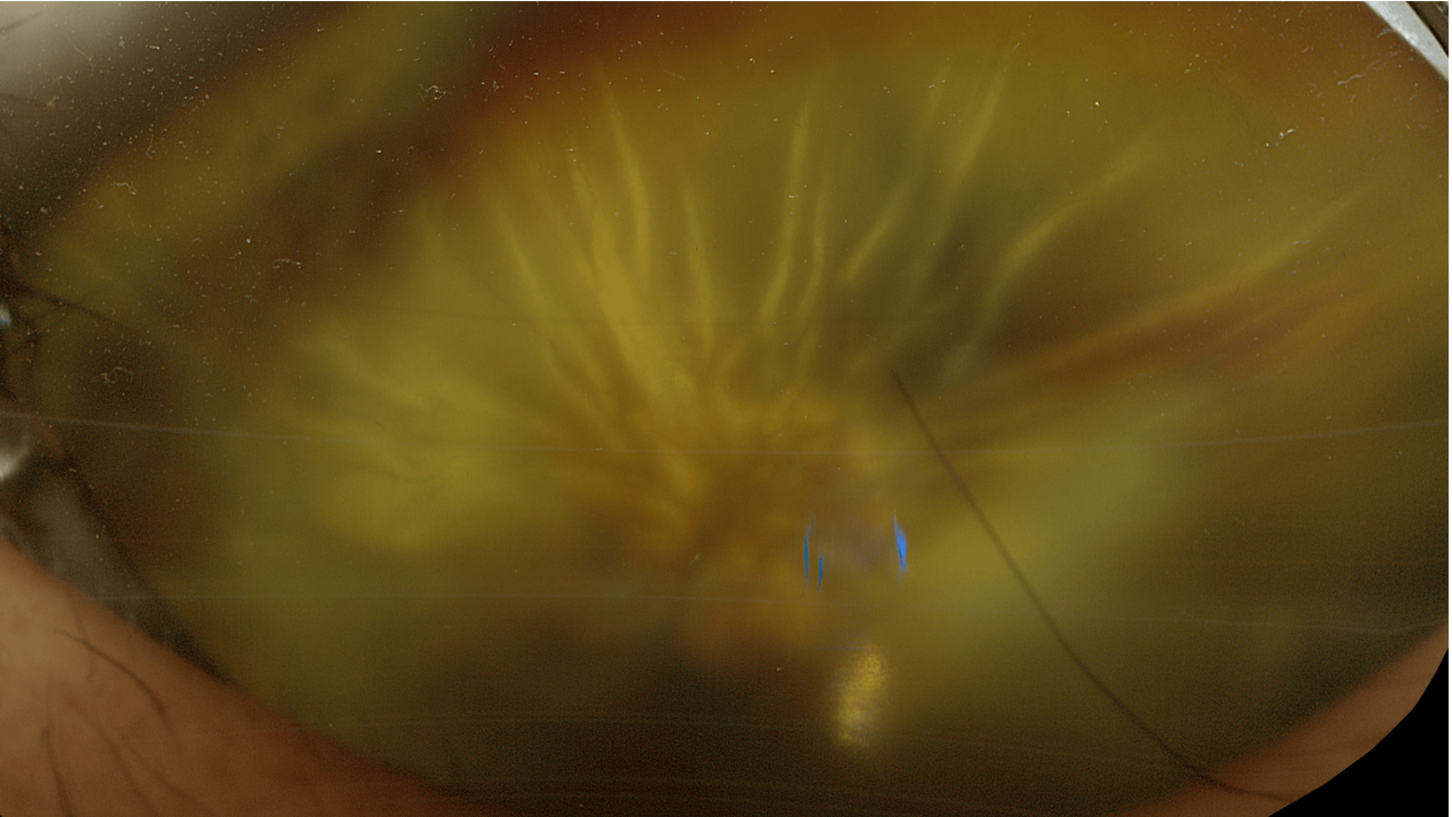
Fundus photograph of the left eye 10 months after Ahmed Glaucoma Valve implantation in the left eye Ultra-widefield fundus imaging (Optos California RGB, Nikon Solutions, Tokyo, Japan) shows total retinal detachment with fixed retinal folds

**Figure 4:**
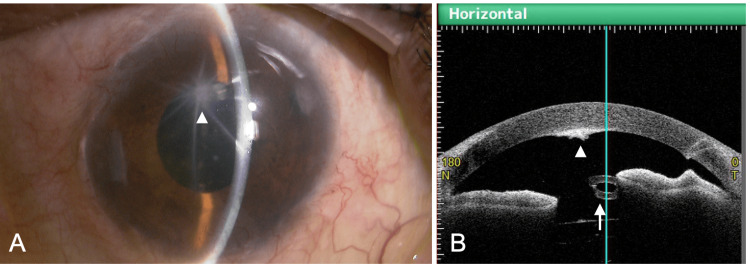
Slit-lamp (A) and anterior-segment OCT (B) findings 10 months after Ahmed Glaucoma Valve implantation in the left eye OCT: optical coherence tomography (A) A stellate corneal opacity is observed. The center of the opacity corresponds to the position of the tube tip (arrowhead). (B) Anterior-segment OCT (CASIA2 Advance, Tomey Corporation, Nagoya, Japan) demonstrates a clear distance between the tube tip (arrow) and the corneal endothelium (arrowhead)

At the 10-month postoperative visit, BCVA OS was no light perception, and IOP was 6 mmHg OS. The patient reported progressive visual loss for more than one month but had not sought medical attention. As this period corresponded to the spring season in Japan, atopic eyelid dermatitis OU worsened, accompanied by severe ocular itching, and the patient frequently rubbed his eyes. Fundus examination revealed total retinal detachment OS, with an inferior peripheral retinal break and fixed retinal folds (Figure 3). At this time, a stellate corneal opacity was observed on the endothelial surface, the center of which corresponded to the tip of the Ahmed tube (Figure 4A, arrowhead). Anterior-segment optical coherence tomography (OCT) (CASIA2 Advance, Tomey Corporation, Nagoya, Japan) showed a clear gap between the tube tip and the corneal endothelium (Figure 4B, arrow and arrowhead), with no substantial change compared with previous visits. Surgical treatment for proliferative vitreoretinopathy was explained, but the patient declined surgery and opted for observation. Intensification of systemic treatment for atopic dermatitis, including biologic agents [13], was also discussed but declined. Only epinastine eye drops OU were continued.

**Figure 5:**
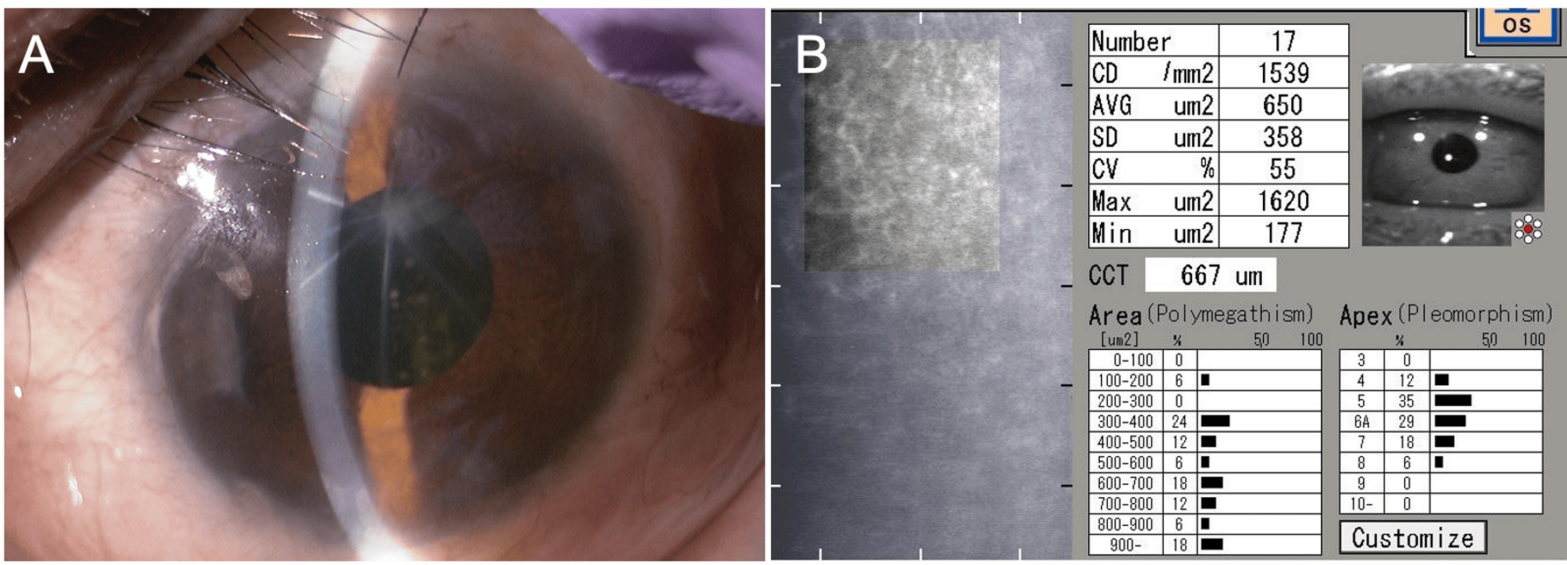
Slit-lamp (A) and specular microscopy (B) findings 16 months after Ahmed Glaucoma Valve implantation in the left eye CD, cell density; AVG, average; SD, standard deviation; CV, coefficient of variation; CCT, central corneal thickness (A) No further progression of corneal opacity is observed. (B) Corneal endothelial cell density has decreased to 1,539 cells/mm², and central corneal thickness has increased to 667 μm

At the final visit (16 months after AGV implantation and six months after detection of corneal opacity), BCVA was 1.2 OD and no light perception OS. IOP was 10 mmHg OD and 9 mmHg OS. With the transition to a cooler season, eyelid dermatitis had partially improved, and no further progression of corneal opacity was observed (Figure 5A). ECD was 1,539 cells/mm², and CCT was 667 μm OS (Figure 5B). The extent of retinal detachment remained unchanged compared with six months earlier. Epinastine eye drops were continued. Throughout the postoperative follow-up period after AGV implantation, the patient’s treatment for atopic dermatitis remained unchanged and consisted of 0.12% betamethasone valerate ointment, 0.05% clobetasol propionate shampoo, 0.5% delgocitinib ointment, and 0.3% heparinoid cream.

## Discussion

In the present case, the location of the corneal endothelial opacity precisely corresponded to the position of the tube tip, suggesting that the inserted tube was involved in the development of the corneal opacity. Notably, at each clinical visit, a sufficient distance between the tube and the cornea was confirmed. In nonvalved glaucoma drainage devices, such as the Baerveldt glaucoma implant, it has been suggested that endothelial damage may occur without direct tube-corneal contact because of aqueous flow directed toward the cornea [14]. However, the AGV has a unidirectional valve mechanism, and the formation of aqueous flow toward the cornea is considered unlikely [14]. In addition, the characteristic configuration of the corneal opacity, with its center corresponding to the tube tip, further supports the possibility that tube contact played a role in its development.

When the tube is inserted into the anterior chamber, Fuchs endothelial dystrophy, iridocorneal endothelial syndrome, and postoperative complications such as hypotony and tube-corneal touch have been reported as risk factors for postoperative corneal endothelial decompensation [9]. Nevertheless, the incidence of tube-corneal touch itself is relatively low [15]. A greater distance between the tube tip and the cornea has been associated with less ECD loss [16]. Therefore, the ciliary sulcus has been proposed as a favorable insertion site during tube shunt surgery in eyes at high risk of tube-corneal touch or corneal decompensation [17]. Accordingly, the occurrence of corneal damage caused by a tube inserted into the ciliary sulcus likely requires additional contributing factors.

In patients with atopic dermatitis, eye rubbing, often performed to alleviate symptoms of atopic keratoconjunctivitis, has been reported to act as a form of self-inflicted trauma and to be associated with various ocular complications [2-6]. The retinal detachment observed in our patient is also considered a typical complication related to eye rubbing [5,6]. Acute corneal hydrops has been reported in patients with atopic dermatitis [2], indicating that corneal deformation caused by eye rubbing can be substantial. In the present case, the timing of corneal opacity development coincided with an exacerbation of atopic dermatitis, and the patient reported frequent eye rubbing. Taken together, intermittent tube-corneal contact induced by corneal deformation during eye rubbing is considered a plausible mechanism underlying the corneal opacity. However, it should be noted that this mechanism remains hypothetical. In this case, an approximately 28% decrease in corneal ECD was observed during follow-up. This finding is also consistent with the possible presence of intermittent tube-corneal contact. It should be noted that proliferative vitreoretinopathy was already present at the time the corneal opacity was detected. Although marked hypotony was not observed during follow-up, the possibility that transient hypotony, shallow anterior chamber, and subsequent tube-corneal contact occurred at some point during the course of retinal detachment cannot be excluded. This case involved a complex clinical background, including chronic inflammation associated with atopic glaucoma, reduced aqueous humor production associated with multiple prior surgeries, and retinal detachment. Therefore, it cannot be excluded that corneal opacity was influenced not by a single mechanism, such as transient tube-corneal contact alone, but by multiple alternative or contributing mechanisms, including transient hypotony, subtle tube movement, or anterior chamber shallowing during retinal detachment.

Although various eye rubbing-related complications have been reported in patients with atopic dermatitis, to our knowledge, there are very few reports that directly address corneal opacity caused by tube contact following glaucoma tube shunt surgery. This case is clinically meaningful in demonstrating that corneal damage related to the tube may occur even after ciliary sulcus tube insertion in patients with atopic glaucoma. Although pars plana tube insertion might have prevented corneal opacity, this approach requires concomitant vitrectomy and may not be a safe option in relatively young patients, as in the present case. Therefore, in patients with atopic glaucoma, appropriate management of atopic dermatitis and adequate control of ocular itching are important not only preoperatively but also throughout the postoperative course.

## Conclusions

In conclusion, this case highlights the possibility that corneal opacity may occur after glaucoma tube shunt surgery with ciliary sulcus tube placement in certain patients with atopic glaucoma. Although unproven, intermittent tube-corneal contact related to eye rubbing and worsening atopic dermatitis may have contributed to the corneal findings. This case highlights the importance of careful postoperative monitoring of corneal status and appropriate, proactive management of atopic dermatitis in patients undergoing tube shunt surgery.
